# Breastfeeding Is Associated with Higher Adherence to the Mediterranean Diet in a Spanish Population of Preschoolers: The SENDO Project

**DOI:** 10.3390/nu15051278

**Published:** 2023-03-04

**Authors:** Asier Oliver Olid, Laura Moreno-Galarraga, Jose Manuel Moreno-Villares, Maria del Mar Bibiloni, Miguel Ángel Martínez-González, Víctor de la O, Alejandro Fernandez-Montero, Nerea Martín-Calvo

**Affiliations:** 1 Department of Pediatrics, Hospital Universitario de Navarra, 31008 Pamplona, Spain; 2 IdiSNA, Instituto de Investigación Sanitaria de Navarra, 31008 Pamplona, Spain; 3 Department of Pediatrics, Clínica Universidad de Navarra, 28027 Madrid, Spain; 4 CIBER Fisiopatología de la Obesidad y Nutrición (CIBEROBN), Instituto de Salud Carlos III (ISCIII), 28029 Madrid, Spain; 5 Research Group on Community Nutrition & Oxidative Stress, University of Balearic Islands, 07122 Palma de Mallorca, Spain; 6 Health Research Institute of the Balearic Islands (IdISBa), 07120 Palma de Mallorca, Spain; 7 Department of Preventive Medicine and Public Health, Facultad de Medicina, Universidad de Navarra, 31008 Pamplona, Spain; 8 Pathophysiology of Obesity and Nutrition, Centro de Investigación Biomédica en Red, Instituto de Salud Carlos III, 28029 Madrid, Spain; 9 Department of Nutrition, Harvard TH Chan School of Public Health, Boston, MA 02115, USA; 10 Department of Occupational Medicine, Clínica Universidad de Navarra, 31008 Pamplona, Spain

**Keywords:** breastfeeding, Mediterranean diet, adherence, childhood obesity, pre-schoolers, diet quality

## Abstract

Objective: To assess whether breastfeeding during the first months of life is associated with adherence to the Mediterranean dietary (MedDiet) pattern in preschool children. Design: The Seguimiento del Niño para un Desarrollo Óptimo (SENDO) project is an ongoing pediatric cohort with open recruitment, started in 2015 in Spain. Participants, recruited when they are 4 to 5 years old at their primary local health center or school, are followed annually through online questionnaires. For this study, 941 SENDO participants with full data on study variables were included. Breastfeeding history was collected retrospectively at baseline. Adherence to the Mediterranean diet was assessed with the KIDMED index (range −3 to 12). Results: After accounting for multiple sociodemographic and lifestyle confounders, including parental attitudes and knowledge about dietary recommendations for children, breastfeeding was independently associated with a higher adherence to the MedDiet. Compared with children who were never breastfed, children breastfed for ≥6 months had a one-point increase on their mean KIDMED score (Mean difference +0.93, 95%confidence interval [CI]. 0.52–1.34, *p* for trend <0.001). The odds ratio of high adherence to the MedDiet (KIDMED index ≥8) was 2.94 (95%CI 1.50–5.36) in children who were breastfed for at least 6 months, as compared to their peers who were never breastfeed. Children who were breastfed for less than 6 months exhibited intermediate levels of adherence (*p* for trend <0.01). Conclusion: Breastfeeding for 6 months or longer is associated with a higher adherence to the Mediterranean diet during the preschool years.

## 1. Introduction

Diet can impact physical, mental, and emotional health. Diet quality among children is a matter of great concern for public health authorities worldwide, as an inadequate diet increases the risk of both undernourishment and obesity, and is associated with overall short- and long-term adverse health outcomes [1,2,3]. A healthy diet needs to provide all the essential macro- and micro-nutrients necessary for children’s growth and development. Nutritional epidemiology is currently focusing more on recommending global food patterns rather than focusing on any specific food or isolated nutrient [4] to better include possible interactions and synergistic effects. In this context, the Mediterranean dietary (MedDiet) pattern is a high-quality food pattern linked to many beneficial health effects [5]. It is characterized by the abundant intake of plant-based foods (vegetables, fruits, legumes, minimally processed grains, and nuts); low consumption of meat; moderate-high consumption of fish; and moderate consumption of dairy products, mainly consumed as yoghurt and cheeses. The global intake of lipids can be high in the MedDiet (around 40% of total energy intake), but the ratio between beneficial monounsaturated and non-beneficial saturated lipids is high, due to the high monounsaturated content of olive oil, which is the main culinary fat used in Mediterranean countries [6,7]. Consistent evidence has reported that the MedDiet is associated with a lower obesity risk o and a lower risk of other non-communicable chronic diseases both in children [8] and adults [9]. 

The World Health Organization (WHO) currently states that breastfeeding is the recommended diet during an infant’s first 6 months of life, and encourages mothers to keep breastfeeding while introducing nutritionally-adequate and safe complementary foods for the first 2 years of age or beyond [10,11]. Breastfeeding provides numerous benefits to both mother and baby, including improved infant health and development, increased maternal bonding, and reduced risk of chronic diseases. Breastfeeding has shown both short- and long-time benefits in infants. In the short term, children who are breastfed show fewer respiratory infections [12], are less irritable, and achieve longer nocturnal sleep [13]. In the long term, children who were breastfed show a lower risk of asthma and atopy [12], obesity [11], cardiovascular disease, hypertension, and type 2 diabetes.

Another positive effect of breastfeeding, is that it has been associated with better diet quality in children, including higher intake of beneficial nutrients such as zinc or iron, and a reduced intake of saturated fats. Recently published articles have reported, for example, that breastfeeding is associated with lower consumption of ultra-processed foods [14] and higher intake of fruit and vegetables [15,16,17]. Along the same lines, a meta-analysis of participants from European cohorts found that the duration of breastfeeding was directly associated with higher consumption of fruits and vegetables in young children [18,19,20]. Therefore, different studies have focused on the relationship between breastfeeding and the dietary intake of different foods and macro- or micro-nutrients [21,22]; however, to our knowledge, whether breastfeeding is associated with certain global food patterns, such as the Mediterranean dietary pattern, has not been yet assessed. 

In this study we aimed to analyze whether breastfeeding was linked to a better adherence to the MedDiet in preschoolers.

## 2. Material and Methods

### 2.1. Study Population

The Seguimiento del Niño para un Desarrollo Óptimo (SENDO) project is an ongoing Spanish pediatric prospective cohort aimed at understanding the impact of diet and lifestyle on children´s health. It is a multipurpose study focused on the prevention of non-communicable diseases, specifically designed to analyze the health benefits of the Mediterranean food pattern in a Mediterranean setting (Spain). Participants are invited to enter SENDO by their pediatrician or by the research team at their school. The recruitment started in 2015 and is permanently open. The current number of participants is 979. Inclusion criteria require being between 4 to 5 years old at recruitment, and living in Spain. The only exclusion criterion is not having access to an internet-connected device to be able to complete the online questionnaires. Information is collected at baseline and updated annually through self-administered online questionnaires mainly completed by legal tutors or parents. The baseline (Q0) questionnaire collects extensive information on participants’ personal and family history, sociodemographic context, anthropometrics, diet, eating behaviors, lifestyles, physical activity, and personality traits. Participants’ data are electronically entered and exported to a secure web-based database

SENDO follows all the rules of the Helsinki Declaration on Ethical Principles for Human Research, and its protocol was approved by the Ethical Committee for Clinical Research of Navarra (Pyto 2016/122). The parents of the participants signed a paper-based informed consent form and mailed it to the study team before entering the SENDO project.

### 2.2. Assessment of the Exposure

Information regarding children´s breastfeeding history, type, and duration was provided retrospectively by the parents through specific questions included in the Basal questionnaire. Parents were asked whether their children had been given any type of breastfeeding (yes/no). Those with an affirmative answer were also asked about the type (exclusive/non-exclusive), the duration of the breastfeeding (less than 1 month, from 1 to <3 months, from 3 to <6 months, from 6 to <12 months, or longer than 12 months), and how old was the baby was when they completely stopped breastfeeding. The information was recategorized for the analyses as duration of total breastfeeding in three categories: never breastfed, breastfed for <6 months, or breastfed for ≥6 months. Non-breastfed children were used as the reference category to provide a baseline against which the effects of breastfeeding could be measured.

### 2.3. Assessment of the Outcome

Dietary assessment tools in SENDO include a food frequency questionnaire (FFQ) and several dietary scoring systems. Adherence to the MedDiet was assessed with the KIDMED (Mediterranean Diet Quality Index for Children) index [23], one of the most frequently used dietary score systems for measuring adherence to the MedDiet in pediatric populations and adolescents. The KIDMED, an a priori-defined dietary index, consists of 16 items, 12 positive items (score 0 or +1), and 4 negative items (score −1 or 0) to assess the intake of different food groups and components of the MedDiet. Thus, the score can be aggregated to provide a total score ranging from −3 to 12 points [24]. Positive items in the KIDMED score include the daily consumption of one or more than one fruit, the daily consumption of one or more than one fresh or cooked vegetable, the consumption of fish (at least 2–3/week), pulses (more than 1/week), pasta or rice (≥5 days/week), nuts (at least 2–3/week), cereal or cereal products and dairy products for breakfast, yoghurts and/or 40 g of cheese daily, and the use of olive oil. Negative items included the consumption of fast food more than once a week, not taking breakfast, using commercially-baked pastries for breakfast, and the daily consumption of sweets and candy. The KIDMED index has been validated in several studies and has been shown to be a reliable tool for assessing adherence to the MedDiet in children. According to their KIDMED score, participants were considered to have poor adherence (≤3 points), medium (4–7 points), or high (≥8 points) adherence to the MedDiet [25,26].

### 2.4. Evaluation of Covariates

A variety of covariates that could potentially impact the results were considered. The baseline questionnaire collected information on children’s factors (gestation, delivery and perinatal information, sociodemographic variables, dietary intake, and different lifestyle factors such as physical activity or sedentary behaviors) and maternal factors (maternal age, race, and education level).

Participant´s body mass index (BMI) was calculated as the ratio of reported weight (kg) to squared height (m^2^). Nutritional status was estimated using sex- and age-specific BMI cut-off points based on the International Obesity Task Force (IOTF) reference standards [27]. Dietary information was collected by a previously-validated semi-quantitative FFQ [28]. A team of dieticians derived the content of nutrients in each element in the FFQ using Spanish food composition tables [29] and online databases [30]. The nutrient content was calculated by multiplying the frequency of intake of each food by the edible serving and the nutrient composition of the specified serving size to calculate the total energy intake.

Information on physical activity was collected using a physical activity questionnaire that included a total of 17 different activities and 10 response categories, ranging from never to 10 or more hours/week. The metabolic equivalents of task (METs)-hour/week for each activity was then estimated by multiplying the number of METs of each activity by the weekly participation in that activity, weighted according to the number of months dedicated to each activity [31]. Total physical activity was quantified by adding the METs-h/week carried out during the free time. Screen time was estimated as the average number of hours dedicated to watching TV, using a computer, or playing video games per day. 

Parental nutrition knowledge and eating attitudes were evaluated using two scores. The parental knowledge of nutritional recommendations for children was assessed with a Nutrition-knowledge score with questions about the recommended consumption frequency of ten food groups (dairy products, fruit, vegetables, cereals, meat, fish, eggs, pulses, nuts, and olive oil). Parents had to choose among 10 categories of response ranging from “never” to “6 or more times a day”. Each question was assigned 1 point if it complied with the dietary recommendations, and 0 points if not. The score was then expressed as a percentage, with a higher value meaning better knowledge about nutritional recommendations for children. For analysis, participants were categorized as low (<40%), moderate (40–70%), or high knowledge (>70%). Low knowledge was used as the reference category. The parents healthy-eating attitudes towards their child’s habits were assessed with an eight-item questionnaire. Each question was given 1 point if it complied with dietary recommendations and 0 points if not; thus, the score ranged from 0 to 8 points. For analysis, parental eating attitudes were categorized into three categories: neglected or unhealthy (from 0 to 3 points), average or moderate (from 4 to 5 points), and positive or healthy eating attitude (from 6 to 8 points). The lowest category was also used as reference. 

### 2.5. Statistical Analysis

Participants’ characteristics were compared by categories of breastfeeding. For descriptive purposes, we used means and standard deviations (SD) for quantitative variables and percentages (%) for categorical variables. Generalized mixed models to account for intracluster correlation among siblings were used. 

We first calculated the mean change in the KIDMED score associated with breastfeeding. Then, we calculated the odds ratio (OR) and 95% confidence interval (CI) for medium-high adherence to the Mediterranean diet using never breastfed as the reference category. We calculated crude- and multivariable-adjusted estimates through 3 progressively adjusting models. The first model was adjusted for sex, age, race (categorized as white vs. others), screen time (continuous), physical activity (continuous), and BMI z-score (continuous). The second model was adjusted for all the variables in model 1 plus gestational age (less than 38 weeks, 38 to 40 weeks, or more than 40 weeks), method of delivery (vaginal or caesarean), birth weight (continuous), maternal high education (yes or not), and maternal age (continuous). Finally, the third model was also adjusted for parental attitudes towards their child’s dietary habits (neglected, average, or healthy) and parental knowledge about nutritional recommendations for children (low, medium, or high). Finally, we calculated the marginal effect of breastfeeding, this is, the adjusted difference (and 95% CI) in the proportion of children with medium-high adherence to the Mediterranean diet between categories of breastfeeding.

## 3. Results

From 979 participants recruited in the SENDO project, 941 children with full data on study variables and recruited between January 2015 and June 2022 were included for final analysis (mean age 5.01 y., SD: 0.85, 51% males). The most relevant demographic characteristics of the 941 study participants according to breastfeeding categories are shown in Table 1. A total of 84.1% of the participants had been breastfed (including all types and durations of breastfeeding) and 54.4% had been breastfed for 6 months or more. Children who were breastfed for longer were more often born by vaginal delivery and had a higher birthweight. Regarding lifestyles, children who were breastfed reported less screen time, and mothers who breastfed their children for longer time were slightly younger and had longer gestations. In our study, maternal education was associated with breastfeeding and mothers who breastfed their children for a longer time showed better knowledge about children’s dietary recommendations and presented healthier attitudes towards their child’s dietary habits.

Using the KIDMED index, a score under 3 reflects poor adherence to the Mediterranean diet, a score from 4 to 7 describes an average adherence, and a score ranging from 8 to 12 reflects high adherence The study participants showed a median score of 6.0 points. The mean KIDMED index was 5.5 in children who were never breastfed, 5.7 in those who were breastfed for less than 6 months, and 6.4 in those who were breastfed for over 6 months. The proportions of children with high adherence to MedDiet (KIDMED ≥8 points) were 11.9% among those who were never breastfed, 20.2% among those breastfed for less than six months, and 26.6% for those who were breastfeed for six months or longer.

We found that those children who were breastfed for six months or longer had a significantly higher KidMed index score compared to those who were never breastfed, with an adjusted average increase of nearly one more point in their final KIDMED index (mean difference +0.93 points (95% CI 0.52 to 1.34) after controlling for all the above-mentioned potential confounders (Table 2). 

**Figure 1:**
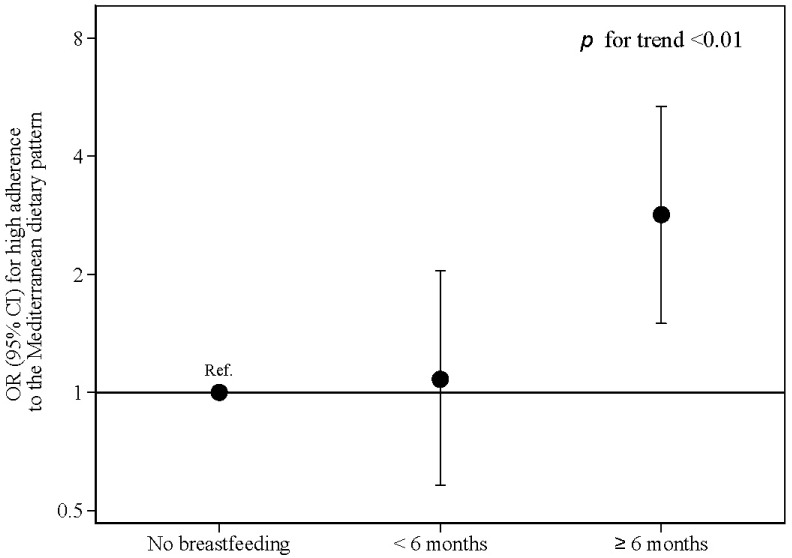
Odds ratio (OR) and 95% confidence interval (CI) for high adherence to the Mediterranean dietary pattern (KIDMED score ≥ 8) associated with breastfeeding history. The SENDO project.

When the KIDMED score was dichotomized (high vs. low), we observed that compared with children who were not breastfed, those who were breastfed for six months or longer had over twofold higher odds (2.63, 95% CI 1.46 to 4.71) of showing high adherence to the MedDiet (Figure 1) in the crude model. The estimates were consistent in progressively adjusted models. The final multivariable model (fully covariate-adjusted model), which controlled for a range of potential confounding variables, showed that breastfeeding for at least 6 months was associated with almost threefold higher odds (2.94, 95% CI 1.50 to 5.36) of high adherence to the MedDiet (*p* for trend <0.01) independently of the child age, sex, race, physical activity, screen time, BMI, gestational age, birth method, and birthweight, as well as maternal age, educational level, dietary knowledge, and attitudes towards their child’s dietary habits. After adjusting for all the previously mentioned confounding factors, compared to the category of children who had not been breastfed, in the category of children who had been breastfed for 6 months or more, we found 9.8% (95% CI: 3.1% 16.3%) (*p* = 0.04) more participants with moderate-high adherence to the MedDiet (Table 3).

Multi adjusted model: Adjusted for sex, race (white or others), screen time (continuous), physical activity (continuous), BMI z score (continuous), gestational age (>38 weeks, 38 to 40 weeks, or more than 40 weeks), way of delivery (vaginal or caesarean), birth weight (continuous), maternal high education (yes or not), maternal age (continuous), parental attitudes towards child’s dietary habits (negative, medium or healthy), and parental knowledge about nutritional recommendations for children (low, medium, or high).

## 4. Discussion

Our study of Spanish preschoolers found a positive linear trend between breastfeeding and adherence to the MedDiet. Furthermore, children who were breastfed for six months or longer showed threefold higher odds of having high adherence to the MedDiet than their peers who were never breastfed. Breastfeeding has been previously associated with lower consumption of ultra-processed foods and higher consumption of fruits and vegetables or specific nutrients, but to our knowledge, this is the first study reporting a direct association between breastfeeding and a healthier overall dietary pattern such as the MedDiet.

Breastfeeding rates vary depending on several factors; in our study, the proportion of children who had been breastfed was slightly higher than the one reported by the Spanish Association of Pediatrics with data of the National Institute of Statistics (around 70% at 6 weeks and 45% at 6 months) [32], but similar to the one found in previous cohort studies [33]. This finding points to the well-known fact that in cohort studies there is self-selection that leads to the sample being composed of participants who are particularly health-conscious and tend to have better adherence to healthier lifestyles [34]. The proportion of children with high adherence to the MedDiet was similar to the results presented in previous studies with Spanish children [35] or children from other Mediterranean countries [36,37].

We observed significant differences in MedDiet adherence in preschoolers who had been breastfed for at least six months. The lack of significant results for those who were breastfed for less than 6 months (compared with those who were not breastfed) may be due to a suboptimal sample size. Nevertheless, we observed a significant linear trend, which suggests a dose-response relationship between the length of the breastfeeding and the odds of having better adherence to the MedDiet in childhood. This, together with the biological plausibility and consistency already discussed, suggests that the reported association may represent a true biological effect. The mechanism that links breastfeeding with later dietary habits is not fully understood. Together with the method of delivery [38], breastfeeding is known to influence the colonization of gut microbiota, which may have some effect on food tolerance or acceptance [39]. Moreover, previous studies suggested that early exposure to different flavors through breastfeeding, impacted by maternal diet, may influence a child’s acceptance of different foods [40,41].

It is important to address possible confounders mediating in this association. An association between breastfeeding and socioeconomic status may, for example, act as a potential confounder. At an ecological level, breastfeeding is more frequent in low income countries, where access to baby formulas is limited [42]. At the individual level, however, breastfeeding is more often observed in highly educated mothers with medium to high economic status [43]; the mother’s type of job and her company’s policy regarding maternal leave may also influence both the initiation and the duration of breastfeeding [44]. Moreover, women who choose to breastfeed tend to follow healthier diets, have better dietary knowledge, and promote an overall healthier diet in their children [45], encouraging consumption of fruits and vegetables and limiting their children’s exposure to unhealthy aliments. However, in our study, the association between breastfeeding and the MedDiet remained significant after the adjustment for maternal age, maternal education level, parental knowledge of children’s dietary recommendations, and parental attitudes towards children’s dietary habits, which suggest that breastfeeding may be an independent predictor of a healthier dietary pattern in childhood. 

Our findings are interesting because they also reinforce the importance of breastfeeding to enhance healthy dietary habits later in life. Previous evidence, including a recently published article from the SENDO project [46], found that preterm children and those born by cesarean were at higher risk of being obese, and both prematurity and cesarean delivery have been associated with lower prevalence of breastfeeding initiation [47]. Along with this association, we observed significantly lower proportions of preterm- and cesarean-delivery children in the category of participants with longer breastfeeding duration. In this scenario, our results may be of value because they can help pediatricians and public health professionals to direct their efforts to actively promote breastfeeding in children at higher risk. We also consider it important to address this association in future studies aimed to analyze the relationship between breastfeeding and long-term health outcomes. In the future, when analyzing these kinds of long-term associations, researchers should consider adjusting their analysis for children’s dietary intake, as it is known to be influenced by breastfeeding duration and therefore could act as a confounder.

We must recognize some study limitations. First, the information used was self-reported by participants’ parents. Previous validation studies in the SENDO project have shown high correlations and excellent agreement in parent-reported data, proving that information reported by SENDO parents such as birthweight, birth length, and anthropometric measures at recruitment was valid to be used in epidemiological research [48]. Information on breastfeeding was collected retrospectively, so it is also susceptible to memory bias and errors, as it requires the recall of past experiences and behaviors. Nevertheless, we consulted the medical records of a random sample of 188 children and observed a 96,8% agreement in breastfeeding history. In those with an affirmative answer (N = 97), we observed a 73.2% agreement in the duration of the breastfeeding. Moreover, as the validity of the self-reported information on breastfeeding was not associated with the child’s adherence to the MedDiet, in case of an information bias, it would lead to a non-differential misclassification; therefore, in any case, it would bias the estimate through the null, making it more difficult to observe statistically significant differences, but not affecting the validity of the results found. Second, the SENDO cohort is mainly composed of highly educated white families and therefore, it is not representative of the Spanish population. Although this factor may affect the generalizability of our findings, it could also have some positive effects, such as a higher validity of the self-reported information and a reduction in the potential confounding caused by heterogeneous socioeconomic variables [49]. Thirdly, although there is a delay between breastfeeding and the quality of the diet when the children are five years of age, the data for both times was collected similarly and is subject to the limitations of cross-sectional design. We also note that recall bias and social factors may provide better results for both breastfeeding and adherence to the MedDiet. Prospective studies are needed for causality to be inferred. Lastly, although our results were robust through the progressively adjusted models, we cannot totally remove the possibility of residual confounding by some non-considered variables.

## 5. Conclusions

In conclusion, we found that infant breastfeeding is directly associated with a healthier diet quality among preschool children, understood as a higher adherence to the MedDiet. This finding was independent of sociodemographic and lifestyle confounders, including parental attitudes and knowledge about dietary recommendations for children. Children who were breastfed for at least six months had a significant increase in their KIDMED score, compared to those who were never breastfed. Furthermore, the odds of high adherence to the Mediterranean diet were nearly three times higher in children breastfed for at least six months compared to those who were never breastfed. 

Public health efforts should be made to follow the World Health Organization breastfeeding recommendations and to encourage mothers to breastfeed for the first six months of life in pursuit of long-term benefits for children’s dietary habits. This study provides additional evidence to support the importance of breastfeeding and its potential benefits on children’s dietary habits, highlighting the importance of early-life interventions to promote healthy dietary habits.

## Figures and Tables

**Table 1:** Demographic and Basal characteristics of study participants according to breastfeeding. The SENDO project.

		Never Breastfed	Breastfed <6 Months	Breastfed ≥6 Months	*p* for Trend
N = 941		150	279	512	
Children’s characteristics					
Sex (% boys)		46.7	47.0	51.4	0.49
Age (years)		5.1 ± 1.0	5.0 ± 0.9	5.0 ± 0.8	0.64
Race (% white)		96.0	97.1	95.9	0.75
Birth weight (kg)		3.1 ± 0.6	3.2 ± 0.5	3.3 ± 0.5	<0.01
Z score of BMI		−0.0 ± 1.2	0.1 ± 1.1	0.1 ± 1.2	0.30
Screen time (hours/day)		1.5 ± 1.1	1.4 ± 1.1	1.2 ± 1.2	<0.01
Physical activity (METs-h/w)		38.5 ± 29.3	42.9 ± 33.7	41.4 ± 29.0	0.36
Gestational age (% <38 weeks)		8.0	4.1	2.3	0.01
Type of delivery (%caesarean)		39.8	26.7	20.7	<0.01
Parental’s characteristics					
Maternal age (years)		35.4 ± 4.6	34.5 ± 4.0	34.7 ± 4.2	0.01
Knowledge about dietary recommendations for children					<0.01
	% Low	31.7	27.9	19.1	
	% Medium	62.7	55.9	67.2	
	% High	5.6	16.2	13.6	
Attitudes towards child’s dietary healthy habits					<0.01
	% Negative	7.0	7.7	3.6	
	% Average	35.2	32.7	30.2	
	% Healthy	57.7	59.6	66.2	
Maternal education (% high) 49.3 55.2 58.2					<0.01

Numbers are mean and standard deviation (± SD) or frequency (%). *p* for trend was calculated for the 3 categories of breastfeeding. Knowledge about dietary nutritional recommendations for children was categorized as low (<40%), moderate (40–70%), or high (>70%). Parental healthy-eating attitudes towards their child’s dietary habits was categorized as negative or unhealthy (0 to 3 points), average or moderate (4 to 5 points), and positive or healthy (6 to 8 points).

**Table 2:** Mean difference and 95% confidence interval (CI) in the KIDMED score by breastfeeding history. The SENDO project.

	Never Breastfed	Breastfed <6 Months	Breastfed ≥6 Months	*p* for Trend
N = 941	150	279	512	
Crude model	0 (Ref.)	0.18 (−0.23 to 0.58)	0.83 (0.48 to 1.19)	
*p* value		0.39	<0.001	<0.001
Model 1	0 (Ref.)	0.13 (−0.27 to 0.52)	0.75 (0.40 to 1.11)	
*p* value		0.53	<0.001	<0.001
Model 2	0 (Ref.)	0.21 (−0.23 to 0.66)	0.86 (0.46 to 1.26)	
*p* value		0.35	<0.001	<0.001
Model 3	0 (Ref.)	0.23 (−0.23 to 0.69)	0.93 (0.52 to 1.34)	
*p* value		0.33	<0.001	<0.001

KIDMED Score was used as a continuous variable, (range −3 to 12). Model 1 is adjusted for sex, race (white or others), screen time (continuous), physical activity (continuous), and z score of the BMI (continuous). Model 2 is adjusted for variables in model 1 and gestational age (26 to 36 weeks, or 36 to 40 weeks, or more than 40 weeks), way of delivery (vaginal or caesarean), birth weight (continuous), maternal high education (yes or not), and maternal age (continuous). Model 3 is adjusted for variables in model 2 and parental attitudes towards child’s dietary habits (negative, average, or healthy), and parental knowledge about nutritional recommendations for children (low, medium, or high).

**Table 3:** Odds ratio (OR) and 95% confidence interval (CI) for high adherence to the Mediterranean dietary pattern (KIDMED score ≥ 8) associated with breastfeeding history.

	No Breastfeeding	Breastfeeding <6 Months	Breastfeeding ≥6 Months	*p* for Trend
N	150	279	512	
High MedDiet adherence (%)	11.9%	20.2%	26.6%	
Crude model	1 (Ref.)	1.09 (0.62 to 1.94)	2.63 (1.46 to 4.71)	
*p* value		0.76	<0.01	<0.01
Model 1	1 (Ref.)	1.03 (0.57 to 1.84)	2.46 (1.35 to 4.48)	
*p* value		0.92	<0.01	<0.01
Model 2	1 (Ref.)	1.05 (0.56 to 1.94)	2.90 (1.53 to 5.49)	
*p* value		0.88	<0.01	<0.01
Model 3	1 (Ref.)	1.08 (0.58 to 2.04)	2.94 (1.50 to 5.36)	
*p* value		0.80	<0.01	<0.01

Model 1 is adjusted for age, sex, race (white or others), screen time (continuous), physical activity (continuous) and z score of the BMI (continuous).Model 2 is adjusted for variables in model 1 and gestational age (26 to 36 weeks, or 36 to 40 weeks, or more than 40 weeks), way of delivery (vaginal or caesarean), birth weight (continuous), maternal high education (yes or not) and maternal age (continuous).Model 3 is adjusted variables in model 2 and parental attitudes towards child’s dietary habits (neglected, average or healthy) and parental knowledge about nutritional recommendations (low, medium, or high).

## Data Availability

Available upon request from the Department of Preventive Medicine and Public Health, School of Medicine, University of Navarra, Pamplona, Spain.
